# An assessment of the airborne longevity of group A Streptococcus

**DOI:** 10.1099/mic.0.001421

**Published:** 2024-01-05

**Authors:** Henry P. Oswin, Evie Blake, Allen E. Haddrell, Adam Finn, Shiranee Sriskandan, Jonathan P. Reid, Alice Halliday, Anu Goenka

**Affiliations:** 1 School of Chemistry, University of Bristol, Cantock’s Close, Bristol, UK; 2 School of Cellular and Molecular Medicine, University of Bristol, Bristol, UK; 3 Paediatric Immunology and Infectious Diseases, Bristol Royal Hospital for Children, Bristol, UK; 4 NIHR Health Protection Research Unit in Healthcare-associated Infection and Antimicrobial Resistance, Imperial College London, London, UK; 5 Centre for Bacterial Resistance Biology, Imperial College London, London, UK

**Keywords:** aerosol, group A streptococcus, transmission, airborne, droplet

## Abstract

A corrigendum of this article has been published full details can be found at 10.1099/mic.0.001549.

Group A streptococcus (GAS) infections result in more than 500 000 deaths annually. Despite mounting evidence for airborne transmission of GAS, little is known about its stability in aerosol. Measurements of GAS airborne stability were carried out using the Controlled Electrodynamic Levitation and Extraction of Bioaerosols onto a Substrate (CELEBS) instrument. CELEBS measurements with two different isolates of GAS suggest that it is aerostable, with approximately 70 % of bacteria remaining viable after 20 min of levitation at 50 % relative humidity (RH), with lower survival as RH was reduced. GAS airborne viability loss was driven primarily by desiccation and efflorescence (i.e. salt crystallization), with high pH also potentially playing a role, given reduced survival in bicarbonate containing droplet compositions. At low enough RH for efflorescence to occur, a greater proportion of organic components in the droplet appeared to protect the bacteria from efflorescence. These first insights into the aerosol stability of GAS indicate that airborne transmission of these respiratory tract bacteria may occur, and that both the composition of the droplet containing the bacteria, and the RH of the air affect the duration of bacterial survival in this environment. Future studies will explore a broader range of droplet and air compositions and include a larger selection of GAS strains.

## Data Summary

Data are available at the University of Bristol data.bris repository, at https://doi.org/10.5523/bris.1cexytjgcqqta2clbsn4qgxqz4

## Introduction

Group A streptococcus (GAS), or *Streptococcus pyogenes*, is estimated by the World Health Organization (WHO) to cause 517 000 deaths annually, of which at least 233 000 are due to rheumatic heart disease (RHD) [1]. The health burden associated with GAS falls most heavily upon children, particularly in low- and middle-income countries, with GAS infection being the most common cause of acquired heart disease in children globally [2,3]. However, whilst there is a clear need to reduce the global disease burden of GAS, effective mitigation strategies remain elusive, in part due to limitations in our understanding of GAS transmission.

Inhalation of respiratory droplets is thought to be an important route of GAS acquisition [4]. Viable GAS has been successfully cultured from air samples in hospital wards with scarlet fever cases [5], and a recent study of transmission after GAS outbreaks in UK nursery/school settings identified genotype-matched GAS strains on settle plates placed on high surfaces, suggestive of airborne dispersal [6]. In New Zealand it was found that poor housing conditions, such as overcrowding and damp, increased the risk of GAS infection [7]. Other studies have demonstrated high rates of GAS infection in homeless shelters and nursing homes [4,8, 9]. The likelihood of airborne transmission of GAS raises questions about the longevity of GAS in aerosols. However, whilst the airborne stability of many other bacterial and viral pathogens has been studied using a variety of techniques [10,13], there has been no previous study of the airborne survival of GAS. Soon after their expulsion from the respiratory tract, particles of respiratory fluid begin to evaporate, until the water activity (*a*_w_) of the droplets is at equilibrium with the surrounding relative humidity (RH). Through this equilibration process, aerosolized GAS can be exposed to extremes in solute concentration, pH, oxidation, light, sudden physical changes (e.g. salt crystallization also known as efflorescence) and rapid fluctuations in temperature, all of which may have an impact on viability. It is important to understand the degree to which the hostile airborne microenvironment impacts on the viability of GAS and how this impact is affected by changes in environmental conditions. Doing so will facilitate interpretation of epidemiological studies of transmission and potentially lead to novel strategies for preventing spread, reducing the incidence of GAS infection. For example, during the coronavirus disease 2019 (COVID-19) pandemic, a new wave of research into the transmission of infectious agents provided clinically important information regarding the effectiveness of infection control measures, such as social distancing [14], mask wearing [15] and ventilation [16], for preventing the transmission of severe acute respiratory syndrome coronavirus 2 (SARS-CoV-2).

Here, we report an initial characterization of the airborne survival of GAS, exploring the impact of both environmental conditions such as RH and the composition of the droplets containing the bacteria. The experiments were carried out using the Controlled Electrodynamic Levitation and Extraction of Bioaerosol onto a Substrate (CELEBS) instrument, which has previously been used to study the airborne longevity of *Escherichia coli* [17]*,* mouse hepatitis virus [18], and SARS-CoV-2[19]. CELEBS provides a high degree of control over the conditions experienced by airborne microbes, allowing for the influence of individual parameters to be explored in detail. In addition, the time resolution accessible by CELEBS allows measurements to be made both with relevance to close contact transmission, in which the bacteria are only airborne for a few seconds prior to inhalation, and with relevance to longer distance transmission (often referred to as airborne transmission). The insights provided by these measurements will help develop new strategies to reduce rates of GAS disease.

## Results

### *emm*1 and *emm*83 GAS show similar airborne stability

**Fig. 1.**
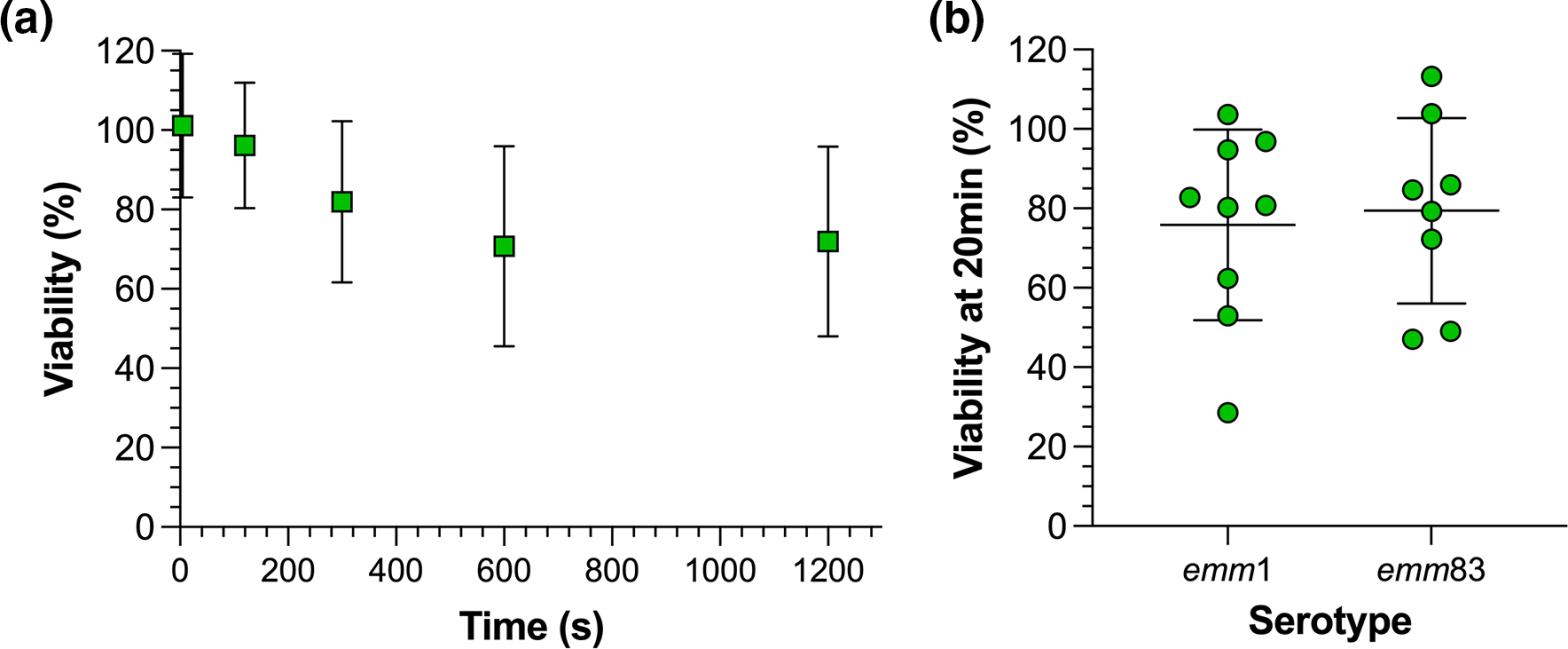
High airborne stability of GAS *emm*1 and *emm*83. CELEBS measurements of GAS % viability in THY broth at 50 % RH comparing the effect of (**a**) varying duration of *emm*1 GAS levitation across mean (standard deviation) of *n*≥3 measurements at 50 % RH. [c.f.u. per droplet at 5 s represents the reference point (100%)] and (**b**) GAS genotypes *emm*1 and *emm*83 after 20 min of levitation. Each datapoint shows the % viability from a single levitation. The central line shows the mean % viability and the error bars show the standard deviation.

Initial characterization of the airborne survival of a clinical isolate of *emm*1 GAS associated with scarlet fever was carried out over the course of 20 min at 50 % RH, 18 °C in droplets of Todd-Hewitt broth with yeast extract (THY broth) (Fig. 1a). These environmental conditions were chosen as they reflect typical indoor conditions. These levitations were carried out in THY broth, the medium used to culture the bacteria prior to levitation, allowing survival to be measured without any additional manipulation. The average number of colony-forming units (c.f.u.) of GAS per droplet remained stable for the first 2 min, but then slowly began to fall, reaching an average of 70 % of the initial number (termed ‘% viability’) at 10 min. Between 10 and 20 min of levitation, no loss of viability was observed, with mean viability remaining 72 % at the latter time point. The % viability of *emm*1 GAS was compared to that of a clinical isolate of *emm*83 GAS associated with necrotizing fasciitis. There was no significant difference between the airborne stability of single *emm*1 and *emm*83 isolates in THY broth at 50 % RH, with both retaining 70–80 % viability after 20 min of levitation (Fig. 1b).

### Droplet composition and relative humidity influence the airborne viability of GAS

It is difficult to reproduce the composition of respiratory fluids in airborne longevity measurements and droplets of various growth media readily available in laboratory settings or artificial simulants of respiratory fluids are often used instead. Respiratory mucosal secretions contain a complex mixture of inorganic salts, proteins and other macromolecules, human cells and bacteria. This composition is influenced by various factors, such as where in the respiratory tract the droplet originates, the health of the person producing the droplet [20] and how much an individual is salivating at the time of droplet generation [21]. As such, if airborne survival measurements are to be applied to the understanding of real-world disease transmission, the influence of variation in droplet composition on survival must be studied.

**Fig. 2.**
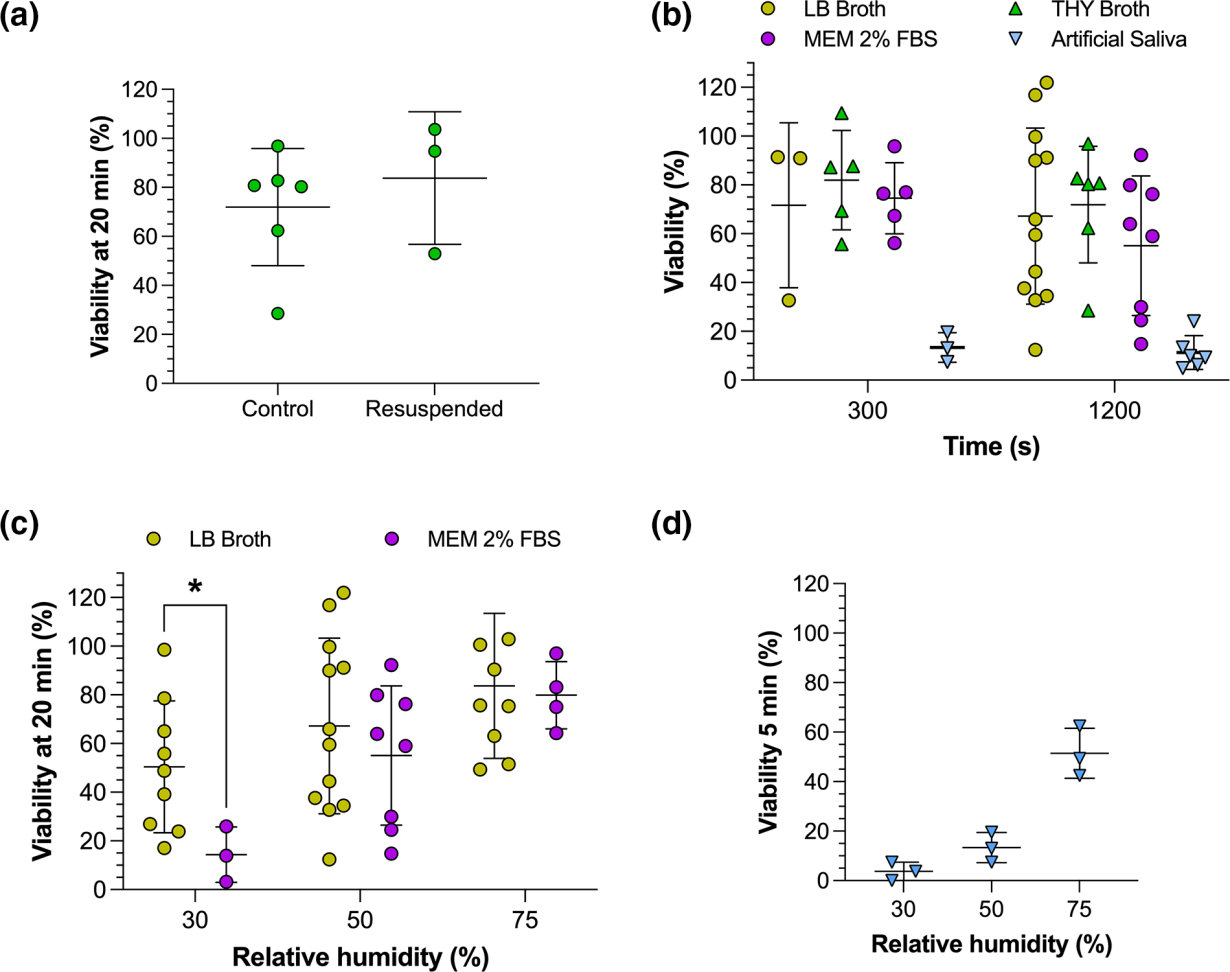
Droplet composition and RH impact on GAS airborne longevity. CELEBS measurements of GAS *emm*1 survival to compare the effect of (**a**) direct loading of THY broth culture into CELEBS (control) or following centrifugation and suspension in fresh THY broth prior to levitation (resuspended) for 20 min at 50 % RH; (**b**) droplet composition in LB broth, THY broth, MEM 2 % FBS, and artificial saliva after 5 and 20 min of levitation at 50 % RH; (**c**) differing RH in LB broth or MEM 2 % FBS, at 30, 50 and 75 % RH after 20 min of levitation; (**d**) artificial saliva in differing RH, at 30, 50 and 75 % RH after 5 min of levitation. Each datapoint shows the % viability from a single levitation. The central line shows the mean % viability and the error bars show the standard deviation. Significance determined by Student’s *t*-test, where *P<0.05.

Studying the viability of GAS in different droplet compositions required centrifugation and resuspension of the bacteria in the various media. Therefore, before different droplet compositions could be studied, it was necessary to understand whether this process affected airborne survival measurements. In THY broth, this was not associated with a significant change in the survival of *emm*1 after a 20 min levitation at 50 % RH (Fig. 2a), suggesting that centrifugation and resuspension of the bacteria in the absence of any change in medium composition had no effect. To study the influence of droplet composition on GAS airborne longevity, GAS *emm*1 was levitated at 50 % RH, 18 °C, for 5 and 20 min in droplets composed of each of four different media: Luria-Bertani (LB) broth, THY broth, Minimum Essential Medium (MEM) 2 % foetal bovine serum (FBS), and artificial saliva [22] (Fig. 2b). Under these conditions no significant changes in bacterial survival between the different media were observed except in artificial saliva, in which it fell to 13 % after 5 min of levitation with little further decay observed between 5 and 20 min. Droplets suspended in a lower RH environment become smaller and contain higher concentrations of solute, which may impact on the viability of pathogens contained within them. To explore this, GAS *emm*1 was levitated for 20 min in either LB broth or MEM 2 % FBS at three different RHs: 30, 50 and 75 % (Fig. 2c). In both media, GAS followed the same trend previously observed for other bacteria and viruses [19,23,26], with the lowest survival at 30 % RH and the highest at 75 % RH. The trend was less apparent in LB broth due to the larger variability between the measurements, but nonetheless viability was significantly lower at 30 than 75 % RH (*P*<0.05). Bacterial survival was significantly lower in MEM 2 % FBS (14 %) than LB broth (50 %) when RH was 30 % (*P*<0.05). To measure the effect of RH on survival in artificial saliva, measurements were carried out at 5 rather than 20 min, as the more rapid decay in artificial saliva made it difficult to see an influence of RH at later time points (Fig. 2d). As for the other media, increased RH resulted in increased airborne survival of GAS in artificial saliva, with an average survival of 4 % at 30 % RH and 51 % at 75 % RH.

### Droplet composition influences efflorescence driven airborne loss of viability in GAS

**Fig. 3.**
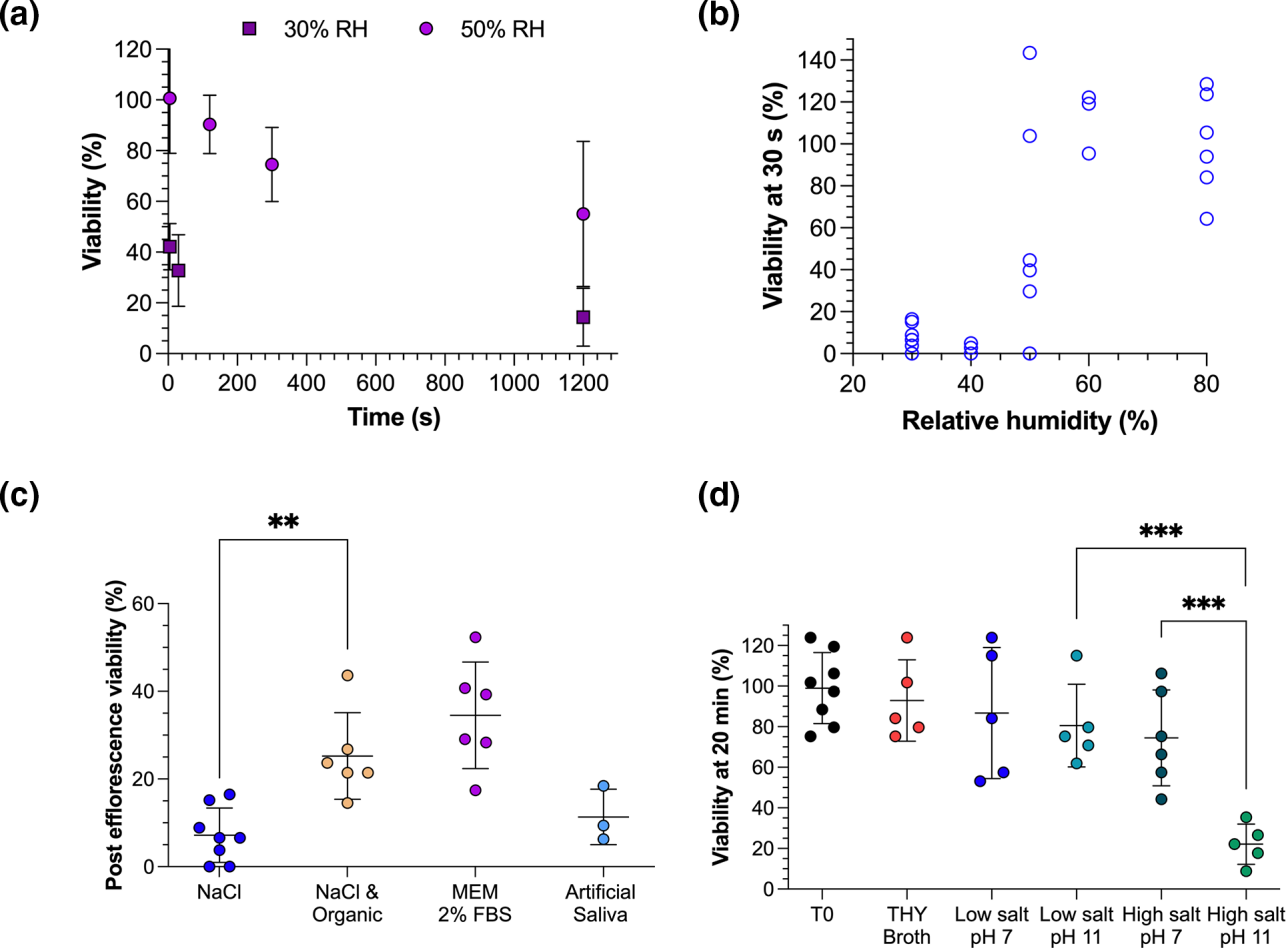
Efflorescence, desiccation and high pH drive the airborne loss of viability of GAS. GAS % viability was compared for (**a**) different RH over time with mean (standard deviation) of *n*≥3 CELEBS measurements at 30 % RH and 50 % RH in droplets of MEM 2 % FBS; (**b**) RH at 30 s of levitation with individual CELEBS measurements in droplets of 10 g l^–1^ NaCl at varying RH; (**c**) varying droplet composition with CELEBS measurement in 10 g l^–1^ NaCl, 10 g l^–1^ NaCl with 1 g l^–1^ glucose and 2 % FBS (organic), MEM 2 % FBS and artificial saliva after 30 s of levitation at 30 % RH; and (**d**) varying salt and pH composition after 20 min of suspension in non-aerosolized bulk solutions of THY broth, 10 g l^–1^ NaCl (labelled low salt) or 350 g l^–1^ NaCl pH 7 (labelled high salt) at pH 7 or pH 11, with T0 indicating the concentration of the culture diluted and spread onto agar with no 20 min incubation step. In (c) and (d) each datapoint shows the % viability from a single measurement and the mean is indicated by central line with standard deviation error bars. Significance determined by Student’s *t*-test, where **P≤0.01, ***P≤0.001.

We next sought to understand the mechanisms driving loss of GAS at low RH. When airborne at a low enough RH, salt-containing droplets will undergo a process termed efflorescence, during which the salts rapidly crystallize. When levitated in MEM 2 % FBS, an immediate loss of viability was observed at 30 % RH, with an average viability of GAS *emm*1 of 42 % after 5 s (Fig. 3a). This is a similar decrease to the previously reported efflorescence-driven infectivity loss that occurs when SARS-CoV-2 is levitated at low RH in MEM 2 % FBS [19]. We therefore hypothesized that under these conditions, efflorescence also drives a loss of viability in GAS *emm*1. To explore this, GAS *emm*1 was levitated for 30 s at varying RH in droplets of water containing 10 g l^–1^ sodium chloride (Fig. 3b). Efflorescence-driven loss of viability was observable, with the viability of GAS *emm*1 falling below 10 % in 30 s when the RH was below the efflorescence threshold (typically 40–50 % for pure NaCl in water) but remaining at 100 % when the RH was above the efflorescence threshold. The measured loss of viability was notably more scattered at 50 % RH, at which we would expect efflorescence to occur more sporadically, with some droplets crystallizing and others not. The loss of viability observed in the pure NaCl droplets was greater than in the MEM 2 % FBS droplets.

When MEM effloresces, part of the resulting particle is crystalized salt and part is organic material such as glucose and protein. We hypothesized that the fraction of bacteria surviving efflorescence in MEM are contained within this organic fraction and it is the absence of this organic material that causes the higher loss of viability in the pure salt droplets. To explore this, levitations for 30 s were carried out at 30 % RH with various droplet compositions (Fig. 3c). When glucose and FBS were added to the NaCl suspension, such that the salt : organic ratio was similar to that of MEM 2 % FBS, the efflorescence event resulted in a significantly lower loss of viability than in pure NaCl droplets (*P*<0.01). The viability only decreased to a mean of 25%, which was closer to the loss observed during efflorescence of MEM 2 % FBS. This supports the hypothesis that increased organic content in the droplet protects GAS from efflorescence-driven loss of viability. However, whilst artificial saliva also contains a high ratio of organic-to-inorganic material, the post-efflorescence viability of GAS in artificial saliva was 11%, which was not significantly different to the loss that occurred in pure salt droplets. Rather than FBS and glucose, the organic fraction in artificial saliva is mucin and pure albumin, suggesting that these organic components may not have the same protective effect as the FBS and glucose in MEM.

### A combination of desiccation and high pH contribute to GAS airborne viability loss

Determining the mechanisms driving airborne losses of viability can be challenging, with airborne survival measurements not always allowing for the relative contribution of different factors to the death of the organism to be disentangled. However, comparison of measurements made in a bulk phase chosen to recreate airborne conditions can allow for an isolation of the impact of some physicochemical parameters on the bacteria specific to airborne droplets. Whilst the water activity and solute concentrations at low RHs are not accessible in bulk solutions, water activities of 0.76 (equivalent to droplets equilibrated to 76 % RH) can be achieved in bulk solutions of sodium chloride. The pH of airborne droplets can also fluctuate, with bicarbonate contained in solutions such as MEM or artificial saliva rapidly partitioning from the droplet into a gas phase at ambient CO_2_ concentration and driving an increase in pH to as high as pH 11 [19,27, 28]. High pH is also readily accessible in bulk solutions.

To determine whether pH and water activity contribute to GAS airborne viability loss, bacteria were resuspended in bulk solutions with varying salt concentration and pH and the viability was measured after 20 min (Fig. 3d). For all but one of the solutions, the viability of the bacteria remained high throughout the 20 min. Whilst high pH (~11) alone did not significantly lower the viability after 20 min, a small decrease in viability was observed in the high-salt solution (350 g ^l–1^ NaCl), with the viability falling to 74 %. This is similar to the average viability after 20 min of levitation at 50 % RH in THY and LB droplets, perhaps suggesting that the desiccation of the bacteria alone is sufficient to drive the airborne loss of viability observed with these droplet compositions. However, the largest loss (84 %) of viability was observed when GAS was suspended in a solution with both high salt and high pH, a significantly larger loss than observed with either high pH or high salt concentration alone (*P*≤0.001). It is possible that the synergistic effect of both low water activity and high pH drives the more rapid losses of viability seen in droplets of MEM and artificial saliva, both of which contain bicarbonate and are expected to become alkaline upon aerosolization [19]. For the airborne survival measurements with droplet compositions other than artificial saliva, the survival after 20 min was higher than seen in the high-salt, high-pH bulk measurement, despite the water activity of the droplets being even lower. This could indicate that the pH of the droplets in CELEBS did not increase to as high as 11. However, it is also possible that the synergistic effects of high pH and desiccation do not occur to the same degree at lower *a*_w_, or that there is a protective effect from other components in the droplet.

## Discussion

The lack of data describing the airborne stability of GAS represents a gap in our understanding of its exposure and transmission. Here, we provide the first data describing the airborne stability of GAS and how it varies with RH and droplet composition. Our results indicate that humid environments could facilitate greater transmission of GAS, which is supported by epidemiological evidence showing damp living conditions as a risk factor for GAS disease [29]. At humidities greater than 70%, it appears that 50–80 % of exhaled GAS remain viable for at least 5 min (Fig. 2), whilst at humidities below the efflorescence threshold of the droplet, a rapid composition-dependent loss of viability can be expected (Fig. 3). These data provide further support for approaches to reduce GAS airborne transmission through better provision of warm and dry living environments. ‘Airborne’ transmission is often not acknowledged as a route of GAS transmission, but these data indicate a need to revisit this paradigm, with the high airborne survival of GAS suggesting that long distance transmission by exhaled aerosol is possible. This is especially the case when coupled with recent evidence indicating a dominant role of airborne transmission in GAS outbreaks in UK nurseries and schools [6], and previous observations of settle plates becoming positive when placed in a room with GAS carriers [30,31]. This suggests that improved ventilation could provide another tool in reducing GAS transmission.

The composition of exhaled respiratory droplets is highly variable and so, rather than recreating the exact composition of respiratory fluids during experimental measurement of airborne survival, it becomes necessary to investigate the potential impact this variation might have. In this study it was identified that the survival of GAS was highly dependent on droplet composition for RH <50 % (Fig. 3c). The observed differences may stem from the apparent influence of droplet composition on efflorescence-driven losses of viability, which appeared to be reduced by the presence of certain organic components in the droplet. This could have relevance for real-world disease transmission as the concentration of salt in human saliva varies significantly and is affected by stimulation of the salivary glands [21], potentially introducing significant variation into the transmissibility of GAS. At 50 % RH and above, the survival appeared to vary less with droplet composition, with artificial saliva being the only formulation that differed significantly (Fig. 2b). The lower overall survival in artificial saliva could be a result of its lower total solute concentration resulting in faster initial evaporation and smaller equilibrated droplet size (a final radius of 4.86 µm at 50 % RH [32], compared to 9.9 µm for MEM 2 % FBS [19] and 8.29 µm for LB broth [17]). A link between equilibrated droplet size and airborne survival has previously been demonstrated for a relatively aerostable strain of *E. coli* (MRE162) [26].

Comparison of our CELEBS measurements with GAS to those we previously carried out with *E. coli* under the same conditions [26] serves to further highlight the relative aerostability of GAS. GAS exhibited an average viability of 67 % after 20 min of levitation at 30 % RH in LB broth droplets (Fig. 2c), compared with 9 % for a relatively aerostable strain of *E. coli*, MRE162 [26]. Oxidative stress is a factor contributing to the airborne decay of *E. coli* in LB broth. It is therefore possible that GAS is more tolerant of reactive oxygen species than *E. coli*. It is also possible that this is simply a difference between Gram-positive and Gram-negative bacteria, a theory that could be explored by expanding CELEBS measurements to cover a broader range of bacterial species. Furthermore, evolutionary adaptation of bacteria to airborne survival may vary according to their individual niche, with bacteria colonizing the respiratory tract such as GAS demonstrating higher airborne survival compared to bacteria such as *E. coli* that do not typically inhabit the respiratory tract.

CELEBS measurements have also previously characterized the airborne survival of SARS-CoV-2 [19], allowing direct comparison to GAS (see Fig. 3a for GAS survival under the same conditions as SARS-CoV-2). SARS-CoV-2 is now widely accepted to spread through the air, yet the airborne stability of SARS-CoV-2 was lower than that of GAS in MEM 2 % FBS droplets levitated at high RH, falling to only 13 % of its initial infectivity in 20 min. However, at low RH in MEM droplets, GAS and SARS-CoV-2 displayed very similar survival profiles, both immediately falling to approximately 40 % viability and then remaining more stable. The similar survival of GAS and SARS-CoV-2 in effloresced MEM suggests that whilst the mechanism of efflorescence-driven loss of viability may vary between microbes, it is a sufficiently forceful process to prevent adaptations that confer survival to an airborne pathogen. It could be assumed from these measurements that GAS is more transmissible than SARS-CoV-2, but there are many factors aside from aerostability that contribute to a pathogen’s airborne transmissibility. For example, SARS-CoV-2 may compensate for its lower aerostability through more rapid replication in the respiratory tract, increasing the concentration of pathogen per exhalation. A low aerostability leading to a 90 % greater loss of infectious pathogen would be offset if the initial concentration in exhaled droplets was 10-fold higher.

Future work will expand upon these first measurements of the airborne stability of GAS. Whilst these measurements could be construed to indicate a similar degree of airborne stability in *emm*1 and *emm*83 GAS, the comparison was limited to single strains. The comparison should be expanded over a wider range of conditions, times, *emm* types and substrains. Levitations for several hours should be carried out to explore how various conditions influence the propensity for GAS to accumulate in poorly ventilated spaces. The influence on airborne survival of varying the growth conditions of the bacteria prior to levitation should be explored, with a particular focus on factors relevant to clinical transmission. Further exploration of the influence of droplet composition on GAS airborne stability should be carried out, aiming to explore the impact of increasingly more realistic droplet formulations. Suspending the bacteria in non-pooled human saliva samples could allow for the influence of interpersonal variability in saliva composition on GAS airborne survival to be studied. Increased mucin concentration has been demonstrated to delay the efflorescence-driven loss of infectivity in MHV [33] and it is possible that this same delay takes place for GAS. It is likely that GAS is present in exhaled droplets with other microbes and understanding the potential influence of interspecies airborne competition on airborne stability remains an entirely unexplored topic of research, which CELEBS is well suited to exploring. Whilst work with bacteria in CELEBS thus far has only explored airborne changes in survival, future work could also examine airborne changes in gene expression and in the capacity of bacteria to infect cells. Through a more complete understanding of the complexities of airborne transmission it is hoped that improved infection control measures may be developed and the global health burden of pathogens such as GAS may be reduced.

## Methods

### Strains and growth conditions

Clinical isolates of *Streptococcus pyogenes* strains H305 (*emm*1 serotype) [34] associated with scarlet fever and BHS0570 (*emm*83 serotype) associated with necrotizing fasciitis were obtained from the Imperial College London strain collection. Bacteria were typically grown overnight at 37 °C, 5 % CO_2_, in THY broth (fat-free minced meat infusion 10 g l^–1^, tryptone 20 g l^–1^, glucose 2 g l^–1^, sodium bicarbonate 2 g l^–1^, sodium chloride 2 g l^–1^, disodium phosphate 0.4 g l^–1^, yeast extract 2 g l^–1^, dissolved in distilled water). Cultures were incubated for 16–20 h, until their OD_600_ reached 1.0, prior to measurement.

### Measuring viability in aerosols

If the bacteria were to be levitated in droplets of a media other than THY broth, the culture was first centrifuged at 2400 ***g*** for 5 min and the pellet resuspended in the desired medium. The bacterial suspension was then diluted to OD_600_ 0.5 in the media used to form the droplets. The diluted culture was then loaded into the droplet dispenser, which was aligned with the electrodynamic balance (EDB) in the centre of the CELEBS chamber. Several droplets (typically four per measurement) with a radius of 24–28 µm each, were sprayed through an induction electrode and into the EDB. The charge applied to the droplets by the induction electrode allowed for the droplets to be trapped in an oscillating electric field created by the ring electrodes of the EDB. The LabVIEW software used to control CELEBS changes the frequency of the field over time, which allowed for droplets to be kept in the field whilst they evaporated, and their mass-to-charge ratio changed. This principle has been previously described [35,36].

The atmosphere around the EDB was maintained at room temperature and the desired relative humidity was obtained through an air inlet angled towards the EDB, the humidity of which can be adjusted by altering the ratio of dry to wet air. The RH of the airflow was measured by an RH probe prior to entering the levitation chamber, which was used to ensure that the airflow entering the chamber was within 1 % of the desired RH. A plate containing Columbia agar with 2 % horse blood (Thermo Scientific PB0122A) was kept in a compartment beneath the EDB, and a small amount of liquid THY broth was applied to the surface of the plate. After the droplets had been suspended for the desired length of time, the compartment was opened, causing the droplets to deposit into the broth on the surface of the plate. The plate was then incubated at room temperature for approximately 5 min before the broth was spread evenly across the surface and placed into an incubator overnight at 37 °C, 5 % CO_2_.

### Calculating bacteria per droplet and percentage survival

The number of bacterial colonies on the agar plate for each levitation was counted manually the day after the experiment (after a minimum of 16 h of incubation) to give the colony-forming units (c.f.u.). A camera was positioned above the EDB, allowing the number of droplets trapped for each measurement to be counted and recorded. The c.f.u. per droplet for each levitation was calculated by dividing the total c.f.u. on the plate by the number of droplets deposited onto the plate. For each experiment several five second levitations were carried out, from which a mean is calculated to identify the initial c.f.u. per droplet. The % viability for each levitation is calculated by dividing the c.f.u. per droplet value for that levitation by the c.f.u. per droplet (from that experiment) and multiplying the resulting value by 100.

### Measuring bulk phase changes in viability

Bacterial cultures were grown overnight as previously described. The cultures were then centrifuged for 5 min with a relative centrifugal force of 8200 ***g***. The resulting pellets were then resuspended in the test solutions. The viability of bacteria in the solution over time was assessed by removing 100 µl from the solution at each time point and diluting it into THY broth at a factor of 10^6^. The number of bacteria within this solution was then assessed by c.f.u. counting. The c.f.u. counts were normalized to a measurement taken at the start of the experiment to allow for values to be expressed in terms of percentage survival.

### Statistical analysis

For each comparison, an F-test was used to determine if the variance of the two datasets was equal. Depending on the results of the F-test, *P* values were calculated using Student’s *t*-test accounting for either equal or unequal variance. In the case of multiple comparisons to a control or comparisons between timecourse datasets an analysis of variance (ANOVA) was first carried out to test for significance, followed by multiple *t*-tests with alpha values adjusted using the Bonferroni–Holm correction for multiple comparisons.

## Abbreviations

CELEBS: controlled electrodynamic levitation and extraction of bioaerosols onto a substrate
c.f.u.: colony-forming units
GAS: group A streptococcus
LB broth: Luria-Bertani broth
MEM: minimum essential medium
RH: relative humidity
RHD: rheumatic heart disease
THY broth: Todd-Hewitt broth with yeast extract
