# Corrigendum: An assessment of the airborne longevity of group A Streptococcus

**DOI:** 10.1099/mic.0.001549

**Published:** 2025-03-20

**Authors:** Henry P. Oswin, Evie Blake, Allen E. Haddrell, Adam Finn, Shiranee Sriskandan, Jonathan P. Reid, Alice Halliday, Anu Goenka

**Affiliations:** 1 School of Chemistry, University of Bristol, Cantock’s Close, Bristol, UK; 2 School of Cellular and Molecular Medicine, University of Bristol, Bristol, UK; 3 Paediatric Immunology and Infectious Diseases, Bristol Royal Hospital for Children, Bristol, UK; 4 NIHR Health Protection Research Unit in Healthcare-associated Infection and Antimicrobial Resistance, Imperial College London, London, UK; 5 Centre for Bacterial Resistance Biology, Imperial College London, London, UK

In the published version of this article, there was an omission in the Funding information, as the article does not list the EPSRC funder grant numbers EP/N509619/1, EP/R513179/1.

Previously, the Funding information statement appeared as below:

This work was funded by a Small Grant Award from the European Society of Paediatric Infectious Diseases (A.H./A.G.). A.H. acknowledges support from the Elizabeth Blackwell Institute, funded in part by the Wellcome Trust (grant number 204813/Z/16/Z). H.O. acknowledges support from the Engineering and Physical Sciences Research Council and the Defence Science and Technology Laboratory.

The correct Funding information statement should have appeared as:

This work was funded by a Small Grant Award from the European Society of Paediatric Infectious Diseases (A.H./A.G.). A.H. acknowledges support from the Elizabeth Blackwell Institute, funded in part by the Wellcome Trust (grant number 204813/Z/16/Z). H.O. acknowledges support from the Engineering and Physical Sciences Research Council [EP/N509619/1,EP/R513179/1] and the Defence Science and Technology Laboratory.

The authors apologize for any inconvenience caused.

